# Hafnium metallocene compounds used as cathode interfacial layers for enhanced electron transfer in organic solar cells

**DOI:** 10.1186/1556-276X-7-74

**Published:** 2012-01-09

**Authors:** Keunhee Park, Seungsik Oh, Donggeun Jung, Heeyeop Chae, Hyoungsub Kim, Jin-Hyo Boo

**Affiliations:** 1 Department of Physics, Brain Korea 21 Physics Research Division, and Institute of Basic Science, Sungkyunkwan University, Suwon, 440-746, Republic of Korea; 2 Department of Chemical Engineering, Sungkyunkwan University, Suwon, 440-746, Republic of Korea; 3 School of Advanced Materials Science and Engineering, Sungkyunkwan University, Suwon, 440-746, Republic of Korea; 4 Department of Chemistry and Institute of Basic Science, Sungkyunkwan University, Suwon 440-746, Republic of Korea

**Keywords:** organic solar cell, cathode interfacial layer, metallocene compounds.

## Abstract

We have used hafnium metallocene compounds as cathode interfacial layers for organic solar cells [OSCs]. A metallocene compound consists of a transition metal and two cyclopentadienyl ligands coordinated in a sandwich structure. For the fabrication of the OSCs, poly[3,4-ethylenedioxythiophene]:poly(styrene sulfonate), poly(3-hexylthiophene-2,5-diyl) + [6,6]-phenyl C_61 _butyric acid methyl ester, bis-(ethylcyclopentadienyl)hafnium(IV) dichloride, and aluminum were deposited as a hole transport layer, an active layer, a cathode interfacial layer, and a cathode, respectively. The hafnium metallocene compound cathode interfacial layer improved the performance of OSCs compared to that of OSCs without the interfacial layer. The current density-voltage characteristics of OSCs with an interfacial layer thickness of 0.7 nm and of those without an interfacial layer showed power conversion efficiency [PCE] values of 2.96% and 2.34%, respectively, under an illumination condition of 100 mW/cm^2 ^(AM 1.5). It is thought that a cathode interfacial layer of an appropriate thickness enhances the electron transfer between the active layer and the cathode, and thus increases the PCE of the OSCs.

## Introduction

Organic solar cells [OSCs] have attracted attention due to their unique advantages, such as easy processing, low cost of fabrication of large-area cells, and mechanical flexibility [1]. However, the efficiency of organic solar cells is not sufficient for them to be used commercially. Therefore, many methods, such as treatment and annealing, have been proposed to improve the device performance [2]. Recently, the most efficient OSCs have been fabricated based on the bulk-heterojunction concept, in which conjugated polymers (electron donors) and fullerenes (electron acceptors) form a three-dimensional network with a large area of phase-separation interface. When photons are absorbed by the organic materials, electron-hole pairs with strong binding energy are generated. The excitons subsequently dissociate, forming free carriers, while they diffuse to the interface between the electron donor and the acceptor. Then, these photogenerated holes and electrons transport through the donor and acceptor materials, respectively, toward the electrodes, eventually resulting in a photocurrent [1-3].

One of the key issues in the development of high efficiency OSCs is the need to increase the charge carrier transport between the active layer and the electrode. Metal electrodes have also received attention in this context. This is not surprising considering the experience with organic light emitting diodes, into which LiF was introduced to enhance the solar cell performance [4]. Recently, several approaches involving the insertion of various thin layers, such as Cs_2_CO_3_, have been reported which aim to improve the electron injection properties between the active layer and the electrode in light-emitting devices [5].

In this work, we investigate the photovoltaic properties of OSCs with hafnium metallocene compounds as the cathode interfacial layer. A metallocene compound consists of a transition metal and two cyclopentadienyl ligands coordinated in a sandwich structure. We used poly(3-hexylthiophene) [P3HT] as the electron donor material and [6,6]-phenyl C_61 _butyric acid methyl ester [PCBM] as the electron acceptor to fabricate OSCs. A thin layer of bis-(ethylcyclopentadienyl) hafnium(IV) dichloride [ECHD] was inserted between the active layer and the cathode. The use of a hafnium metallocene compound cathode interfacial layer improved the performance of OSCs compared to that of OSCs without the interfacial layer.

## Experiments

**Figure 1:**
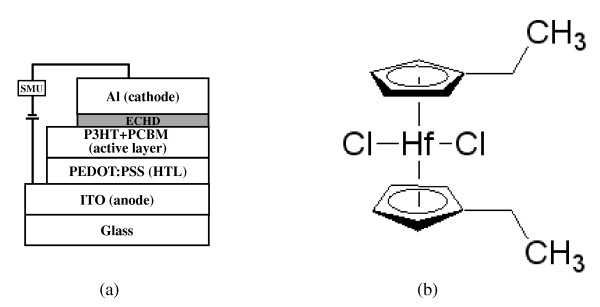
**The structure of the solar cell and the chemical structure of the ECHD**. (**a**) A schematic drawing of an organic solar cell structure with a bis-(ethylcyclopentadienyl) hafnium(IV) dichloride cathode interfacial layer. (**b**) The chemical structure of the bis-(ethylcyclopentadienyl) hafnium(IV) dichloride.

The structure of the solar cell and the chemical structure of the ECHD are presented schematically in Figure 1. To fabricate the OSCs, poly (styrene sulfonate)-doped poly(3,4-ethylene dioxythiophene) [PEDOT:PSS] (26 nm), a mixture of P3HT and PCBM (80 nm), ECHD (various thickness), and aluminum [Al] (80 nm) were deposited on the indium-tin-oxide[ITO] anode as a hole transport layer, a photo active layer, and a cathode, respectively. The substrates used in this study were commercially available ITO-coated glass (Samsung Corning, Corning Inc., Corning, NY, USA) with an ITO film thickness of 1,425 Å and a sheet resistance of 11.1 Ω/sq. First, the ITO glass was cleaned successively in ultrasonic baths of trichloroethylene, acetone, methanol, and deionized water for 10 min each. A mixture of PEDOT:PSS and isopropyl alcohol with a weight ratio of 1:2 was used for spin-coating. A mixture of P3HT and PCBM (P3HT + PCBM) with the optimized weight ratio of 1:1 was prepared with chlorobenzene (4 wt.%). Thin films of PEDOT:PSS and P3HT + PCBM were formed on the ITO-coated glass by spin-coating. The spin speed of the polymer film was 4,000 rpm for PEDOT:PSS and 1,000 rpm for P3HT + PCBM. Then, ECHD and Al were deposited on the P3HT + PCBM film by thermal evaporation. The current density-voltage characteristics were determined by using a solar simulator (Luzchem, LZC-SSR, Keithley 2400 SourceMeter, Kiethley Instruments Inc., Cleveland, OH, USA) under standard conditions of air mass and 100 mW/cm^2 ^(AM 1.5) at room temperature. The absorbance spectra for the films were measured using a UV-Visible [Vis] spectrophotometer (Optizen 2120uvpuls, Mecasys Co., Ltd., Yuseong-gu, Daejeon, South Korea) to determine the influence of the ECHD layer on the absorption of the solar spectrum. The surface roughness was determined by atomic force microscopy [AFM] (ThermoMicroscopes Corporation, Sunnyvale, CA, USA). Spectra were recorded on AXIS NOVA (Kratos Inc., Chestnut Ridge, NY, USA) using a He I (21.22 eV) source for ultraviolet photoelectron spectroscopy [UPS] analysis to investigate the electronic properties of the ECHD/Al structure. UPS spectra were measured with the sample biased at -15 V to clear the detector work function.

## Result and discussion

**Figure 2:**
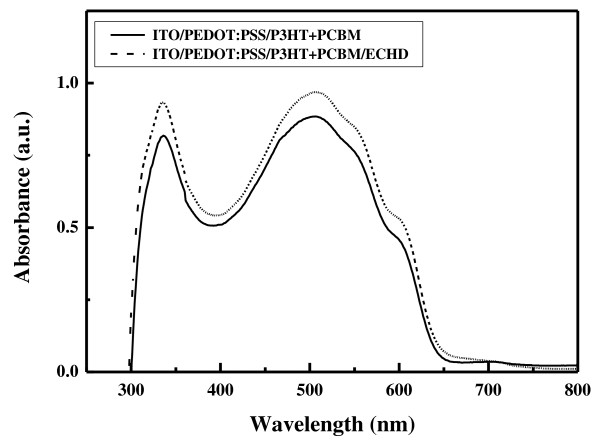
**UV-Vis absorption spectra of the ITO/PEDOT:PSS/(P3HT + PCBM)/ECHD and ITO/PEDOT:PSS/(P3HT + PCBM) structures**.

The absorption spectra of ITO/PEDOT:PSS/(P3HT + PCBM) structures with and without a cathode interfacial layer are shown in Figure 2. Both samples showed good absorption in the visible range. The absorption spectrum of the sample with the ECHD cathode interfacial layer was similar to that without the ECHD layer.

**Figure 3:**
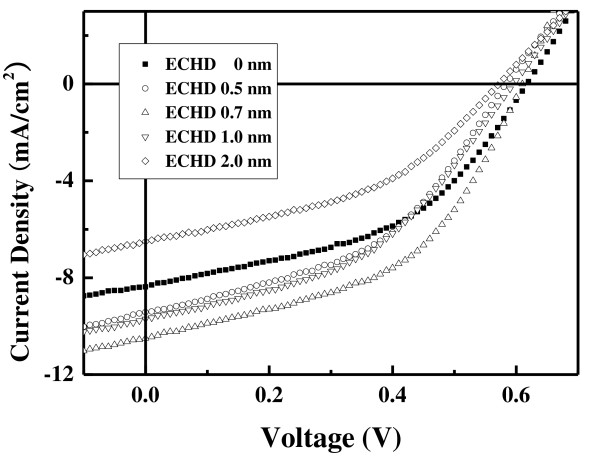
***J*-*V *characteristics of organic solar cells with various thicknesses of the ECHD cathode interfacial layer**. These are taken under an AM 1.5 illumination of 100 mW/cm^2^.

The current density versus applied voltage [*J*-*V*] characteristics of the organic solar cells with various thicknesses of ECHD are shown in Figure 3 under illumination with 100 mW/cm^2 ^(AM 1.5). The device without the interfacial layer was used as the control, and the devices are designated according to the thickness of the ECHD cathode interfacial layer. The thickness of the ECHD cathode interfacial layer was varied between 0.5 nm and 2.0 nm. The values characterizing the photovoltaic performances of the OSCs, such as the short circuit current density [*J*_sc_], open circuit voltage [*V*_oc_], fill factor [FF], and power conversion efficiency [PCE], are given in Table 1. We see that the interfacial ECHD layer at the cathode leads to an increase of *J*_sc _from 8.38 to 10.5 mA/cm^2^. The highest PCE in this set of experiments was 2.96% for the device with an ECHD thickness of 0.7 nm.

**Table 1:** Characteristics of organic solar cells with different thicknesses of the ECHD cathode interfacial layer

OSCs	*J*_sc _(mA/cm^2^)	*V*_oc _(V)	FF (%)	PCE (%)
Control	8.38	0.62	45	2.34
ECHD 0.5 nm	9.43	0.59	45	2.46
ECHD 0.7 nm	10.5	0.61	46	2.96
ECHD 1.0 nm	9.7	0.60	43	2.52
ECHD 2.0 nm	7	0.59	51	1.77

OSCs, organic solar cells; ECHD, bis-(ethylcyclopentadienyl) hafnium(IV) dichloride; *J*_sc_, short circuit current density; *V*_oc_, open circuit voltage; FF, fill factor; PCE, power conversion efficiency.

**Figure 4:**
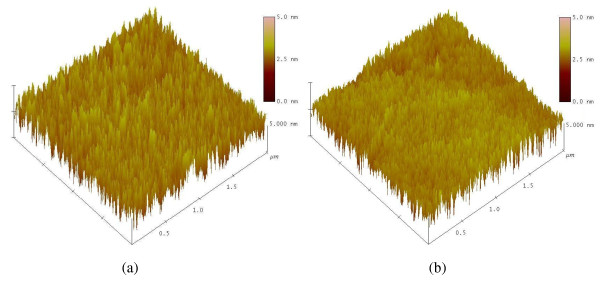
**The AFM images of (a) ITO/PEDOT:PSS/(P3HT + PCBM)/ECHD and (b) ITO/PEDOT:PSS/(P3HT + PCBM)**.

Figure 4 shows the AFM images of the ITO/PEDOT:PSS/(P3HT + PCBM) and ITO/PEDOT:PSS/(P3HT + PCBM)/ECHD structures. The size of the scanned area was 2 μm × 2 μm. For the sample without the ECHD layer, the root mean square [RMS] roughness of the surface was 1.3 nm. However, the sample with the ECHD layer had an RMS roughness of 0.8 nm. The film spikes, which are thought to be caused during the heat treatment after spin-casting, can exist in the P3HT + PCBM active layer. If the metal cathode is directly deposited on to the active layer with the film spikes, an inhomogeneous distribution of the electric field may occur at the P3HT:PCBM/cathode interface. We guess, therefore, that the deposition of an ultrathin cathode interfacial layer prior to the metal cathode deposition may smoothen the interface and leads to a more homogeneous distribution of electric field at the P3HT:PCBM/cathode interface. As a result, when the device is properly biased, a more even electron current will flow between the active layer and the cathode, and higher efficiency can thus be expected as reported by Shrotriya et al. [6].

**Figure 5:**
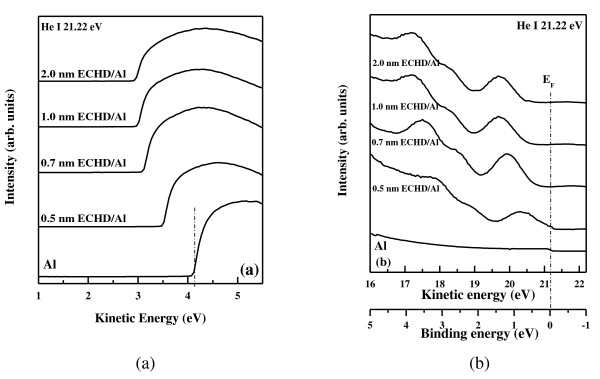
**UPS spectra in the low kinetic and low binding energy regions**. (**a**) UPS spectra in the low kinetic energy region from ECHD/Al structures. The onset of secondary electrons for Al is shown by vertical bars. (**b**) UPS spectra in the low binding energy region from ECHD/Al structures.

Figure 5a shows the UPS spectra at the secondary electron cutoff. The cutoff energies, *E*_cutoff_, of Al and ECHD/Al structures with ECHD thicknesses of 0.5, 0.7, 1.0, and 2.0 nm were found to be 4.12, 3.50, 3.12, 3.07, and 3.07 eV, respectively. It should be noted that the difference between the *E*_cutoff _values of the ECHD/Al structures and that of the Al layer was increased by the insertion of ECHD. Figure 5b shows the UPS spectra of Al and ECHD/Al structures with different ECHD thicknesses. The UPS spectrum of the Al layer around the Fermi edge was shifted to a higher binding energy by the presence of the ECHD layer. All spectra shown in Figure 5b are vertically shifted and plotted using a low scale to clearly display the Fermi edge [7].

**Figure 6:**
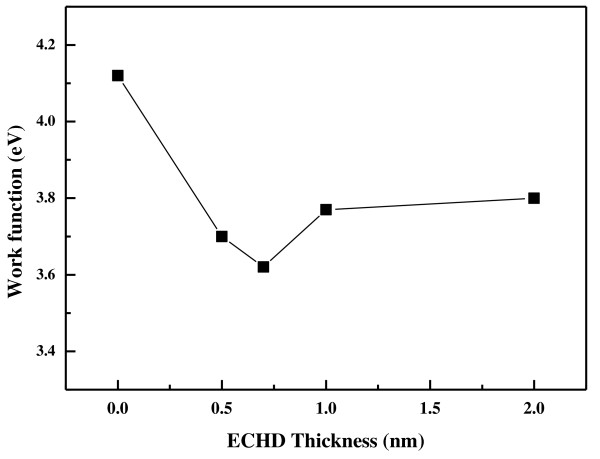
**Changes of work functions in the ECHD/Al structures**. These are measured from UPS measurements as a function of ECHD thickness.

The spectra shown in Figure 5a, b illustrate the relationships between the width of the spectrum, the sample work function *Φ*, and the photon energy *hν*. By subtracting the binding energy of the low energy cutoff from the high binding energy edge of the UPS spectra, the work function of the sample is obtained [8]. The change in the work function of ECHD/Al for various ECHD thicknesses is shown in Figure 6. As the ECHD thickness increased from 0 to 0.7 nm, *Φ *decreased by as much as 0.50 eV. However, further increasing the ECHD thickness above 0.7 nm increased the *Φ *values of ECHD/Al structures. In this experiment, therefore, the minimum *Φ *value was found for the ECHD (0.7 nm)/Al structure. In this structure, the *Φ *value was decreased to 3.62 eV from the *Φ *of Al, which is 4.12 eV.

A possible reason for this decrease of the work function could be due to the hafnium [Hf] element contained in the ECHD layer. The work function of Hf is reported to be 3.9 eV, while Al is reported to have a *Φ *value in the range of 4.06 to 4.26 eV [9]. Such a small *Φ *value of the Hf element compared to that of Al may have contributed to a reduction of the work function of ECHD/Al structure when the thickness of ECHD was increased up to 0.7 nm. It seems that for ECHD layers with thicknesses over 0.7 nm, the *Φ *value of ECHD/Al system has less influence from the Hf element. This finding suggests that an ECHD layer of proper thickness at the Al interface improves electron transport, possibly by lowering the work function of the ECHD/Al structure compared to that of Al, resulting in an enhanced performance of OSCs.

## Conclusion

A metallocene compound (ECHD) that has one hafnium and two cyclopentadienyl ligands coordinated in a sandwich structure was used as a cathode interfacial layer in OSCs. In this study, we demonstrated that ECHD can be utilized as an efficient cathode interfacial layer in OSCs based on P3HT + PCBM. Introduction of the ECHD layer increased the OSC efficiency from 2.34% to 2.96%, possibly resulting from a reduction of the work function, leading to better electron transport at the active layer/Al interface. In our UPS experiment, the minimum work function value of 3.62 eV was found for an ECHD/Al structure with an ECHD thickness of 0.7 nm. It is thought that the smoother surface of P3HT + PCBM with ECHD compared to that of P3HT + PCBM without an ECHD layer also helped to enhance the efficiency.

## Competing interests

The authors declare that they have no competing interests.

## Authors' contributions

The work presented here was carried out in collaboration among all authors. KP, DJ, HC, HK, and JHB defined the research theme. KP and SO carried out the laboratory experiments and analyzed the data. HC, HK, and JHB analyzed the data and discussed the analysis. DJ designed the experiments and discussed the analysis. KP and DJ wrote the manuscript. All authors read and approved the final manuscript.
